# Two-year safety outcomes of iPS cell-derived mesenchymal stromal cells in acute steroid-resistant graft-versus-host disease

**DOI:** 10.1038/s41591-024-02990-z

**Published:** 2024-05-22

**Authors:** Kilian Kelly, Adrian J. C. Bloor, James E. Griffin, Rohini Radia, David T. Yeung, John E. J. Rasko

**Affiliations:** 1 Cynata Therapeutics Limited, Cremorne, Victoria Australia; 2 Haematology & Transplant Unit, The Christie NHS Foundation Trust, Manchester, UK; 3 Department of Bone Marrow Transplantation, University Hospitals Bristol NHS Foundation Trust, Bristol, UK; 4 Department of Haematology, Nottingham University Hospitals NHS Trust, Nottingham, UK; 5 School of Medicine, University of Adelaide, Adelaide, South Australia Australia; 6 Royal Adelaide Hospital, Adelaide, South Australia Australia; 7 Central Clinical School, Faculty of Medicine & Health, University of Sydney, Sydney, New South Wales Australia; 8 Gene and Stem Cell Therapy Program Centenary Institute, University of Sydney, Sydney, New South Wales Australia; 9 Department of Cell and Molecular Therapies, Royal Prince Alfred Hospital, Sydney, New South Wales Australia

**Keywords:** Stem-cell research, Drug development

## Abstract

The first completed clinical trial of induced pluripotent stem cell (iPS cell)-derived cells was conducted in 15 participants with steroid-resistant acute graft-versus-host disease. After intravenous infusion of mesenchymal stromal cells (CYP-001 derived from a clone of human iPS cells), we reported the safety, tolerability and efficacy within the primary evaluation period at day 100. We now report results at the 2-year follow-up: 9 of 15 (60%) participants survived, which compares favorably with previously reported outcomes in studies of steroid-resistant acute graft-versus-host disease. Causes of death were complications commonly observed in recipients of allogeneic hematopoietic stem cell transplantation, and not considered by the investigators to be related to CYP-001 treatment. There were no serious adverse events, tumors or other safety concerns related to CYP-001. In conclusion, systemic delivery of iPS cell-derived cells was safe and well tolerated over 2 years of follow-up, with sustained outcomes up to 2 years after the first infusion. ClinicalTrials.gov registration: NCT02923375.

## Abstract (web summary)

Sustained safety outcomes were seen at 2-year follow-up after induced pluripotent stem cell-derived mesenchymal stromal cell infusion in 15 individuals with steroid-resistant acute graft-versus-host disease.

## Main

While mesenchymal stem cells (MSCs) show potential immunomodulatory activities that could be valuable in the treatment of several diseases, their efficacy in the context of graft-versus-host disease (GvHD), a potentially fatal complication of allogeneic hematopoietic stem cell transplantation (HSCT), has been markedly inconsistent^1^. This variability has been attributed to substantial scalability and manufacturing variations associated with primary donor-derived MSC production, potentially leading to unpredictable medicinal products and suboptimal clinical outcomes.

A fundamental challenge for conventional MSC manufacture lies in the disparity between the small number of MSCs isolated from a single tissue donation and the large number of cells required to treat adults. For example, while a bone marrow collection yields a starting population of approximately 10,000–80,000 MSCs^2^, a typical bone marrow-derived MSC dose regimen requires a total of at least 1 × 10^8^ MSCs for an average adult^3^. Thus, extensive ex vivo culture expansion is necessary to generate enough cells to treat a single patient, with even greater levels of expansion required to manufacture batches of allogeneic MSCs. While such in vitro passaging can generate large numbers of therapeutic doses per donation, it can result in MSCs undergoing functional changes and ultimately entering replicative senescence^2,4^. Consequently, there is a limit to the extent that donor-derived MSC populations can be expanded without adversely affecting cell functionality.

In theory, if the number of therapeutic doses produced per donation could be kept to a minimum, then the need for culture expansion could also be minimized. However, such an approach would necessitate frequent use of new donations, which is problematic given the extent of variability in MSC populations derived from different donors. The immunomodulatory activity of MSCs is in part mediated by the expression of indoleamine 2,3-dioxygenase, which is produced when MSCs are activated by inflammatory cytokines, including interferon-γ (IFNγ) and tumor necrosis factor (TNF). These in turn lead to suppression of T cell proliferation. However, there is a high level of interdonor variability in the propensity of MSCs to be activated by IFNγ and TNF, and thus in their capacity to express indoleamine 2,3-dioxygenase^5–7^. MSC gene expression, differentiation, proliferation and colony-forming capacity are also donor-dependent and tissue source-dependent^2,8^.

The use of iPS cells as a starting material offers an alternative approach to facilitate consistent, large-scale manufacture of MSC-based therapies. iPS cells can replicate indefinitely without loss of pluripotency, in addition to the ability to differentiate into any adult cell type^9–11^. The Cymerus iPS cell-based platform facilitates large-scale production of consistent, allogeneic MSCs from a single cell bank, which in turn was derived from a single blood donation. This approach avoids both interdonor variability and excessive MSC culture expansion. The good manufacturing practice-compliant Cymerus process uses xenogen-free, serum-free and feeder-free conditions to reduce variability within the process and to minimize the risk of contamination with zoonotic agents. Additionally, the process and quality control tests were designed to ensure the absence of residual undifferentiated iPS cells in the final product to avoid the risk of teratoma formation, which is a defining characteristic of undifferentiated pluripotent cells^12^.

The first clinical trial (ClinicalTrials.gov registration: NCT02923375) of Cymerus MSCs (CYP-001) was conducted in adults with steroid-resistant acute GvHD (SR-aGvHD) after an allogeneic HSCT across seven centers in the United Kingdom and Australia^12^. Eligible individuals were required to have been diagnosed with grades II–IV aGvHD followed by steroid resistance in the opinion of the investigator. Steroid resistance was defined as failing to respond or progressing after receipt of a steroid regimen and duration consistent with normal practice at the relevant clinical site (minimum 3 days at a dose of at least 1 mg per kg per day). After providing consent, one individual withdrew before receiving CYP-001, after experiencing a myocardial infarction, and was thus excluded from the analysis. Participants were sequentially assigned to cohort A or cohort B. Participants in cohort A (*n* = 8) received two intravenous infusions of CYP-001, one on day 0 and one on day 7, at a dose of 1 × 10^6^ cells per kg body weight, up to a maximum absolute dose of 1 × 10^8^ cells. Participants in cohort B (*n* = 7) also received infusions of CYP-001 on days 0 and 7, but at a dose of 2 × 10^6^ cells per kg, up to a maximum absolute dose of 2 × 10^8^ cells. In addition to CYP-001, all participants continued treatment with concomitant standard of care aGvHD medications, as reported previously, but were not permitted to receive other investigational agents until at least 28 days after the first dose of CYP-001.

The primary trial evaluation period concluded at 100 days after the first dose of CYP-001. Extended follow-up of up to 2 years required participants to attend clinical assessment visits every 6 months, which consisted of survival status, GvHD grade assessment, details of any additional GvHD treatment received, malignancy status and adverse events.

As reported previously, CYP-001 was safe and well tolerated during the primary evaluation period, with encouraging efficacy outcomes (complete and overall response rates of 53% and 87%, respectively). This represented the first report of safety and efficacy in a completed human clinical trial using iPS cell-derived cells in any disease worldwide. We now report the results of the 2-year follow-up.

No serious adverse events, tumors or other safety concerns related to CYP-001 treatment were identified during the follow-up period.

**Fig. 1:**
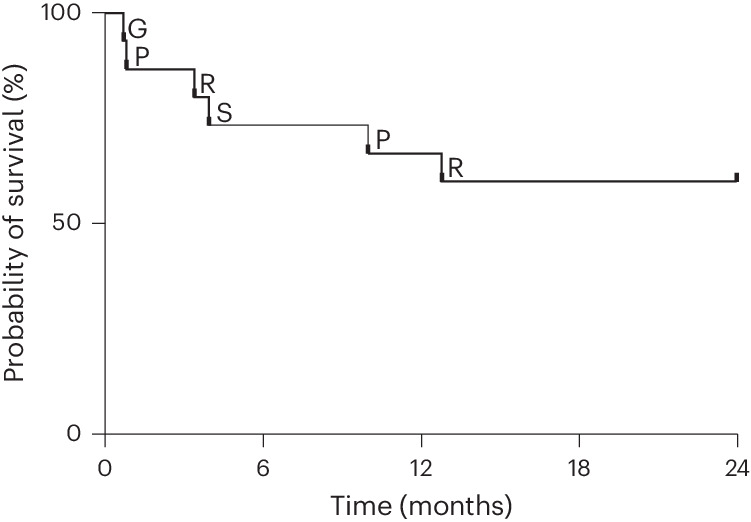
Kaplan–Meier survival curve. Letters represent cause of death: G, GvHD; P, pneumonia; S, sepsis/multi-organ dysfunction; R, relapse. Source data

Nine of the 15 participants (60%) treated with CYP-001 survived for at least 2 years (Fig. 1). Two deaths occurred during the previously reported primary evaluation period and four occurred during the extended follow-up period. None of the deaths was considered by investigators to be related to CYP-001. Reported causes of death were commonly recognized complications observed in recipients of allogeneic HSCT (relapse of preexisting malignancy (*n* = 2); pneumonia (*n* = 2); GvHD (*n* = 1); and sepsis or multi-organ dysfunction (*n* = 1)).

Survival and GvHD status are summarized in Table 1. Three participants exhibited ongoing aGvHD symptoms at the 6-month visit. Two of those participants had grade I aGvHD at the 6-month visit, which represented a partial response in both cases (at baseline, the participants had grades II and III aGvHD, respectively). Another participant had grade II aGvHD at the 6-month visit, which represented stable disease (this participant had grade II aGvHD at baseline and every visit up to 6 months, but was free of GvHD at the 12-month, 18-month and 24-month visits). No participant had aGvHD symptoms at 12 months or later. Three participants had chronic GvHD (cGvHD) at the 12-month and 24-month visits, and two participants had cGvHD at the 18-month visit. Participants who developed cGvHD received additional treatment, including corticosteroids, calcineurin inhibitors, protein kinase inhibitors, mycophenolate mofetil and extracorporeal photopheresis.

**Table 1:** Survival and GvHD status at the extended follow-up visits

Status	6 months	12 months	18 months	24 months
Survival	11/15 (73%)	9/15 (60%)	9/15 (60%)	9/15 (60%)
No GvHD	8/11 (73%)	6/9 (67%)	7/9 (78%)	6/9 (67%)
Grade I aGvHD	2/11 (18%)	0/9 (0%)	0/9 (0%)	0/9 (0%)
Grade II aGvHD	1/11 (9%)	0/9 (0%)	0/9 (0%)	0/9 (0%)
Grade III aGvHD	0/11 (0%)	0/9 (0%)	0/9 (0%)	0/9 (0%)
Grade IV aGvHD	0/11 (0%)	0/9 (0%)	0/9 (0%)	0/9 (0%)
cGvHD	0/11 (0%)	3/9 (33%)	2/9 (22%)	3/9 (33%)

Caution must be exercised when comparing outcomes from different clinical trials, but if the 60% 2-year overall survival rate in participants treated with CYP-001 is confirmed in larger studies, it would compare favorably with previously reported outcomes in patients with SR-aGvHD. For example, several studies of MSCs from other tissue sources in SR-aGvHD reported 2-year overall survival rates ranging from 0% to 40% (refs. ^13–19^). Additionally, the Janus kinase inhibitor ruxolitinib has been approved for the treatment of SR-aGvHD by multiple regulatory authorities, including the US Food and Drug Administration^20^. A pivotal phase III study in patients with SR-aGvHD reported encouraging ruxolitinib response rates (complete response: 34%; overall response: 62%). However, 2-year overall survival could not be evaluated and overall survival at 18 months was 38% in the ruxolitinib group and 36% in the ‘best available therapy’ control group (which involved treatment with antithymocyte immunoglobulin, extracorporeal photopheresis, MSCs, low-dose methotrexate, mycophenolate mofetil, everolimus, sirolimus, etanercept or infliximab)^21^. Furthermore, a recent study of ‘real-world’ experience with a bone marrow-derived MSC product reported that the probability of overall survival in adults with ruxolitinib-refractory aGvHD after MSC treatment at 6, 12 and 24 months was 47% (38–56%), 35% (27–44%) and 30% (22–39%), respectively^22^.

In conclusion, CYP-001 was safe and well tolerated in this study, with sustained outcomes at the planned 2-year follow-up. A global phase II clinical trial of CYP-001 in aGvHD (ClinicalTrials.gov registration: NCT05643638) commenced in 2023.

## Methods

The study was designed, implemented and reported in accordance with the International Conference on Harmonization Harmonized Tripartite Guidelines for Good Clinical Practice, with applicable local regulations and with the ethical principles laid down in the Declaration of Helsinki version 2013. The protocol was approved by the North East-York Research Ethics Committee, Jarrow, United Kingdom, on behalf of all participating centers in the United Kingdom (reference no. 16/NE/0316) and the Royal Adelaide Hospital Human Research Ethics Committee, Adelaide, Australia, on behalf of both participating centers in Australia (reference no. HREC/16/RAH/412). Prospective participants were required to provide written informed consent before screening. The manufacture and quality control of CYP-001 and the design and conduct of the primary evaluation period of the clinical trial (ClinicalTrials.gov registration: NCT02923375) were as described previously^12^. Surviving participants who completed the 100-day primary evaluation period then attended follow-up visits at 6, 12, 18 and 24 months after the initial infusion of CYP-001.

During the follow-up period, efficacy was assessed on the basis of overall survival and GvHD status, while safety was assessed on the basis of serious adverse events, including death and malignancy status. Clinical trial data were collected using ClinCapture v.2.1.15.15 Rev. 3743. Data were analyzed using SAS v.9.4. The Kaplan–Meier survival curve was generated using Prism 10 (GraphPad Software).

### Reporting summary

Further information on research design is available in the Nature Portfolio Reporting Summary linked to this article.

## Online content

Any methods, additional references, Nature Portfolio reporting summaries, source data, extended data, supplementary information, acknowledgements, peer review information; details of author contributions and competing interests; and statements of data and code availability are available at 10.1038/s41591-024-02990-z.

### Supplementary information

Reporting Summary

### Source data

Source Data Fig. 1 Raw survival data for each patient used to create the Kaplan–Meier survival curve. The Kaplan–Meier survival curve was generated using Prism (GraphPad Software), not Excel, but the data are provided in Excel format.

## Data Availability

Researchers may submit a methodologically sound proposal to access raw or analyzed data from 9 months to 36 months after publication, directed to info@cynata.com. Cynata Therapeutics (the study sponsor) will promptly review the request and determine whether the requested data can be shared, and will respond within 8 weeks of receiving the request. Patient-related data were collected as part of a clinical trial and may be subject to patient confidentiality restrictions. Any data that can be shared will be released via a material transfer agreement. Source data are provided with this paper.
