# Supplementary material for: Two-year safety outcomes of iPS cell-derived mesenchymal stromal cells in acute steroid-resistant graft-versus-host disease
Source: Nat Med. 2024 May 22;30(6):1556–8. doi: 10.1038/s41591-024-02990-z (PMC11186752; doi:10.1038/s41591-024-02990-z)
Supplement: Supplementary file 1 — Reporting Summary [file 41591_2024_2990_MOESM1_ESM.pdf]

nature portfolio

Corresponding author(s): John Rasko

Last updated by author(s): Apr 5, 2024

Reporting Summary

Nature Portfolio wishes to improve the reproducibility of the work that we publish. This form provides structure for consistency and transparency in reporting. For further information on Nature Portfolio policies, see our Editorial Policies and the Editorial Policy Checklist.

Statistics

For all statistical analyses, confirm that the following items are present in the figure legend, table legend, main text, or Methods section.

n/a	Confirmed	
☐	☒	The exact sample size (*n*) for each experimental group/condition, given as a discrete number and unit of measurement
☒	☐	A statement on whether measurements were taken from distinct samples or whether the same sample was measured repeatedly
☒	☐	The statistical test(s) used AND whether they are one- or two-sided<br>*Only common tests should be described solely by name; describe more complex techniques in the Methods section.*
☒	☐	A description of all covariates tested
☒	☐	A description of any assumptions or corrections, such as tests of normality and adjustment for multiple comparisons
☒	☐	A full description of the statistical parameters including central tendency (e.g. means) or other basic estimates (e.g. regression coefficient) AND variation (e.g. standard deviation) or associated estimates of uncertainty (e.g. confidence intervals)
☒	☐	For null hypothesis testing, the test statistic (e.g. *F*, *t*, *r*) with confidence intervals, effect sizes, degrees of freedom and *P* value noted<br>*Give P values as exact values whenever suitable.*
☒	☐	For Bayesian analysis, information on the choice of priors and Markov chain Monte Carlo settings
☒	☐	For hierarchical and complex designs, identification of the appropriate level for tests and full reporting of outcomes
☒	☐	Estimates of effect sizes (e.g. Cohen's *d*, Pearson's *r*), indicating how they were calculated

Our web collection on statistics for biologists contains articles on many of the points above.

Software and code

Policy information about availability of computer code

Data collection	Clinical trial: data were collected using ClinCapture V. 2.1.15.15 Rev: 3743.
Data analysis	Data were analyzed using SAS 9.4. The Kaplan Meier Survival Curve was generated using GraphPad Prism 10.

For manuscripts utilizing custom algorithms or software that are central to the research but not yet described in published literature, software must be made available to editors and reviewers. We strongly encourage code deposition in a community repository (e.g. GitHub). See the Nature Portfolio guidelines for submitting code & software for further information.

Data

Policy information about availability of data

All manuscripts must include a data availability statement. This statement should provide the following information, where applicable:

- Accession codes, unique identifiers, or web links for publicly available datasets
- A description of any restrictions on data availability
- For clinical datasets or third party data, please ensure that the statement adheres to our policy

Researchers may submit a methodologically sound proposal to access raw and/or analyzed data from 9 months to 36 months following publication, directed to info@cynata.com. Cynata Therapeutics Limited (the study Sponsor) will promptly review the request and determine whether requested data can be shared, and will respond within 8 weeks of receiving the request. Patient related data were collected as part of a clinical trial and may be subject to patient confidentiality restrictions. Any data that can be shared will be released via a material transfer agreement.

Research involving human participants, their data, or biological material

Policy information about studies with human participants or human data. See also policy information about sex, gender (identity/presentation), and sexual orientation and race, ethnicity and racism.

Reporting on sex and gender	This clinical trial was open to people of any sex and/or gender. The clinical trial enrolled a total of seven participants of female sex and 8 participants of male sex, as reported in the original manuscript on this trial - Bloor et al, Nat Med. 2020:26; 1720–1725. No data was collected on gender (identity/presentation). No sex- or gender-based analyses were performed. Sex and/or gender did not determine eligibility in the clinical trial design, and the sample size was insufficient to facilitate such analyses.
Reporting on race, ethnicity, or other socially relevant groupings	This clinical trial was open to people of any race, ethnicity and other socially relevant groupings. All participants enrolled were white (not hispanic/latino) - as reported in the original manuscript on this trial - Bloor et al, Nat Med. 2020:26; 1720–1725. No analyses based on race, ethnicity and other socially relevant groupings were performed, as the sample size was insufficient to enable such analyses. Race, ethnicity and other socially relevant groupings did not determine eligibility in the clinical trial design, and the sample size was insufficient to facilitate such analyses.
Population characteristics	The study recruited male and female subjects aged between 18 to 70 years (inclusive) who had undergone an allogeneic HSCT to treat a haematological disorder (including but not limited to haematological malignancy) and who had subsequently been diagnosed using consensus grading with Grade II-IV SR-aGvHD (based on the 1994 Consensus Conference on Acute GVHD Grading.
Recruitment	Patients were recruited from seven clinical centres in the United Kingdom (five) and Australia (two) between 10 May 2017 to 28 August 2018. Patients were recruited in accordance with the inclusion and exclusion criteria stipulated in the study protocol, from the relevant site's database or referral. There was no self-selection or other bias likely to be present.
Ethics oversight	The protocol was approved by the North East - York Research Ethics Committee, Jarrow, United Kingdom, on behalf of all participating centres in the United Kingdom (reference number: 16/NE/0316) and the Royal Adelaide Hospital Human Research Ethics Committee, Adelaide, Australia, on behalf of both participating centres in Australia (reference number: HREC/16/RAH/412).

Note that full information on the approval of the study protocol must also be provided in the manuscript.

Field-specific reporting

Please select the one below that is the best fit for your research. If you are not sure, read the appropriate sections before making your selection.

☒ Life sciences ☐ Behavioural & social sciences ☐ Ecological, evolutionary & environmental sciences

For a reference copy of the document with all sections, see nature.com/documents/nr-reporting-summary-flat.pdf

Life sciences study design

All studies must disclose on these points even when the disclosure is negative.

Sample size	As this was an early Phase I safety study no formal sample size was calculated. A sample size of 16 (eight per cohort) was selected in consultation with regulatory authorities, on the grounds that this number of subjects was considered sufficient to evaluate initial safety and tolerability of the product, which was the primary objective of the study.
Data exclusions	No data were excluded from the analyses.
Replication	No replication was performed as this was an open label Phase 1 clinical trial in 15 patients with a rare disease. Further clinical trials will be performed with the objective of confirming the findings.
Randomization	Not applicable. This was an open label Phase 1 clinical trial, in which all subjects received the investigational medicinal product in addition to standard of care treatment, so no randomization was performed.
Blinding	This was an open label clinical trial, in which all subjects received the investigational medicinal product in addition to standard of care treatment.

Reporting for specific materials, systems and methods

We require information from authors about some types of materials, experimental systems and methods used in many studies. Here, indicate whether each material, system or method listed is relevant to your study. If you are not sure if a list item applies to your research, read the appropriate section before selecting a response.

Materials & experimental systems

n/a	Involved in the study
☒	☐ Antibodies
☐	☒ Eukaryotic cell lines
☒	☐ Palaeontology and archaeology
☒	☐ Animals and other organisms
☐	☒ Clinical data
☒	☐ Dual use research of concern
☒	☐ Plants

Methods

n/a	Involved in the study
☒	☐ ChIP-seq
☒	☐ Flow cytometry
☒	☐ MRI-based neuroimaging

Eukaryotic cell lines

Policy information about cell lines and Sex and Gender in Research

Cell line source(s)	The iPSCs used were derived from a fully consented healthy adult human donor, and were reprogrammed using a transgene free, viral-free and feeder-free technique by Cellular Dynamics International, Madison, WI, USA.
Authentication	Short tandem repeat analysis is used to confirm that the cells match the donor.
Mycoplasma contamination	The cell line and differentiated cells used in the final product tested negative for mycoplasma.
Commonly misidentified lines (See ICLAC register)	No commonly misidentified cell lines were used.

Clinical data

Policy information about clinical studies

All manuscripts should comply with the ICMJE guidelines for publication of clinical research and a completed CONSORT checklist must be included with all submissions.

Clinical trial registration	NCT02923375
Study protocol	Full protocol was provided as related manuscript file with previous manuscript on this study (Bloor et al, Nat Med 2020;26:1720-1725)
Data collection	The Christie NHS Foundation Trust, Manchester, UK (open for recruitment from 1 March 2017 - 23 May 2018)<br>Royal Adelaide Hospital, Adelaide, Australia (open for recruitment from 6 March 2017 - 23 May 2018)<br>Nottingham University Hospitals NHS Trust, Nottingham, UK (open for recruitment from 11April 2017 - 23 May 2018)<br>University Hospitals Bristol NHS Trust, Bristol, UK (open for recruitment from 9 May 2017 - 23 May 2018)<br>Leeds Teaching Hospital NHS Trust, Leeds, UK (open for recruitment from 13 May 2017 - 23 May 2018)<br>Sydney Local Health District, Sydney, Australia (open for recruitment from 6 June 2017 - 23 May 2018)<br>Clatterbridge Cancer Centre NHS Foundation Trust (open for recruitment from 16 October - 23 May 2018)
Outcomes	Outcome measures were pre-specified in the study protocol.<br><br>The outcome measures for the follow-up period reported in this manuscript (from Day 100 to 2 years after the first dose of CYP-001) were as follows:<br><br>- Safety, as assessed on the basis of Serious Adverse Events deemed possibly, probably or definitely related to CYP-001, and malignancy status.<br>- Efficacy as assessed on the basis of:<br>- Overall survival at 6, 12, 18 and 24 months.<br>- GvHD status at 6, 12, 18 and 24 months.<br>- Additional GvHD treatment during the follow-up period.

Plants

Seed stocks	N/A
Novel plant genotypes	N/A
Authentication	N/A
